# Real‐world evaluation of teplizumab in type 1 diabetes: Progression to insulin requirement

**DOI:** 10.1111/dom.70354

**Published:** 2025-12-22

**Authors:** Sahana Mahesh, Matthew Anson, Rayaz A. Malik, Daniel J. Cuthbertson, Uazman Alam

**Affiliations:** ^1^ Department of Diabetes, Obesity and Endocrinology University Hospital Aintree, Liverpool University NHS Foundation Trust Liverpool UK; ^2^ Department of Medicine Weill Cornell Medicine‐Qatar, Qatar Foundation, Education City Ar‐Rayyan Qatar; ^3^ Department of Cardiovascular and Metabolic Medicine University of Liverpool Liverpool UK; ^4^ Metabolism & Nutrition Research Group Liverpool University Hospitals NHS Foundation Trust Liverpool UK; ^5^ Liverpool Centre for Cardiovascular Sciences University of Liverpool and Liverpool University Hospitals NHS Foundation Trust Liverpool UK; ^6^ Centre for Biomechanics and Rehabilitation Technologies Staffordshire University Stoke‐on‐Trent UK

**Keywords:** antidiabetic drug, beta cell function, insulin therapy, real‐world evidence, type 1 diabetes

## 1 BACKGROUND

Type 1 diabetes (T1D) is a chronic autoimmune disease, typically diagnosed in childhood and adolescence, characterised by immune‐mediated destruction of pancreatic β‐cells. Multiple immune pathways contribute to this process, including autoreactive CD4+ and CD8+ T‐cells, B‐cells, and innate immune activation, driven by a complex interplay of genetic susceptibility (HLA and non‐HLA loci) and environmental triggers that together define heterogeneity across metabolic and immunogenetic profiles.^1^, ^2^ The resulting insulin deficiency necessitates lifelong insulin therapy. Early‐onset T1D and prolonged insulin use are associated with reduced life expectancy and greater cardiovascular morbidity and mortality.^3^ Preventive strategies are therefore required to delay disease onset.

Teplizumab, an Fc receptor–binding anti‐CD3 monoclonal antibody, was recently approved to delay T1D onset.^4^ It is licensed for use in individuals ≥8 years with Stage 2 T1D, defined by ≥2 positive islet autoantibodies, dysglycaemia without hyperglycaemic symptoms, and insulin use. Previous immunomodulatory therapies raised concerns about feasibility and long‐term immunosuppression.^1^ Teplizumab is the first agent shown to delay clinical disease onset without evidence of chronic immunosuppression.^4^ A meta‐analysis of 8 randomised trials including 754 patients reported that Teplizumab preserved C‐peptide, reduced HbA1c, and delayed insulin initiation by up to 24 months versus placebo.^5^ Common adverse effects include rash, transient lymphopenia, abnormal liver function, gastrointestinal symptoms, and cytokine release syndrome.^6^, ^7^

As clinical trial populations are highly selected, the generalizability of these findings to real‐world populations is uncertain. We therefore aimed to assess the clinical outcomes of patients treated with Teplizumab using electronic health records.

## 2 METHODS

We conducted a retrospective observational cohort study using TriNetX, a global federated research network comprising >150 million patients across >150 healthcare organisations (HCOs).

Patients were eligible if they were aged ≥8 years and had been prescribed Teplizumab. Those with prior insulin use were excluded. Baseline characteristics, including mean glycated haemoglobin (HbA1c), serum C‐peptide, islet autoantibody counts, and oral glucose tolerance test (OGTT) values were evaluated. The index event was the initiation of Teplizumab therapy.

### 2.1 Ethical approval

Formal ethical approval was not required, as only anonymised, aggregated data from the federated TriNetX platform were analyzed. Publication agreements are in place with HCOs.

## 3 OUTCOMES

A treatment‐pathway analysis was performed to identify patients who commenced insulin therapy‐ a proxy marker for progression to (symptomatic) Stage 3 T1D, as TriNetX does not perform stage‐wise coding of T1D diagnoses. An analysis of health outcomes was performed to observe the incidence of drug‐related adverse events (ADEs). *Pre‐specified outcomes were*:

- *Primary outcome*: (a) proportion of patients treated with Teplizumab who progressed to insulin therapy and (b) median time to insulin initiation.
- *Secondary outcomes*: (a) change in HbA1c, (b) change in serum C‐peptide, and (c) incidence of clinically significant ADEs, including rash, lymphopenia, abnormal liver function, and anaphylaxis, defined by ICD‐10 codes.

## 4 STATISTICAL ANALYSIS

Data extraction occurred on 17 November 2025. Statistical analysis for cohort data was performed within the TriNetX platform. Descriptive statistics were used to summarise baseline demographic and clinical variables. Continuous data are presented as median (IQR) or as median (range) as appropriate, and categorical variables as counts and percentages.

Kaplan–Meier survival analysis was used to evaluate time‐to‐event outcomes, specifically the time from teplizumab initiation to insulin commencement. Patients who had relevant outcomes coded before the analysis window or who did not experience the event during follow‐up were censored at their last recorded observation.

## 5 RESULTS

Fourty‐two patients prescribed Teplizumab without prior insulin use were identified.

### 5.1 Baseline biochemical data

Limited data was available. Median age at initiation of Teplizumab was 12 years (IQR 6). Median HbA1C was 5.7% (39 mmol/mol) (IQR 0.1%, *n* = 22) while <10 patients had C‐peptide [median 1.8 (IQR 1)], glutamic acid decarboxylase 65 (GAD‐65) [median 18.6 IU/mL (IQR 84)], and zinc‐transporter 8 (ZnT8) [median 0.14 IU/mL (IQR 1.43)] results (Table 1).

**TABLE 1:** Demographic and clinical characteristics of patients at baseline (*n* = 42).

Characteristic	Teplizumab (*n* = 42)	Range (min, max)
*Demographic details*		
Age at index, years (Median, IQR)	12 (6)	8, 55
Follow up, days (Median, IQR)	197 (357)	
Male sex, no. (%)	25 (60%)	
Race or ethnic group, no. (%)		
White	30 (71%)	
Hispanic or Latino	<10	
Asian	<10	
American Indian or Alaska Native	<10	
Black or African American	0	
*Anthropometric details*		
Body mass index, kg/m^2^ (median, IQR)	21.4 (5.37)	16.2, 38
Body surface area, m^2^ (median, IQR)	1.55 (0.41)	1.33, 5
*Biochemistry*		
Glycated haemoglobin, % (Median, IQR) (*n* = 21)	5.7 (0.4)	(4.9%, 7.3%)
Glycated haemoglobin, mmol/mol (Median) (*n* = 21)	39	30, 56
Oral glucose tolerance test (OGTT), mg/dL (median, IQR) (*n* ≤ 10)	122 (23)	122, 145
C‐peptide, ng/mL (median, IQR) (*n* ≤ 10)	1.8 (0.32)	1, 6.6
T1D associated autoantibodies, IU/mL (median, IQR) (*n* ≤ 10)		
GAD‐65	18.6 (84)	6, 106
IA‐2	8.1 (2.1)	8.1, 10.2
Insulin‐Ab	0 (0)	0, 0.05
Zt‐8	83 (145)	0, 174

### 5.2 Follow up data

**FIGURE 1:**
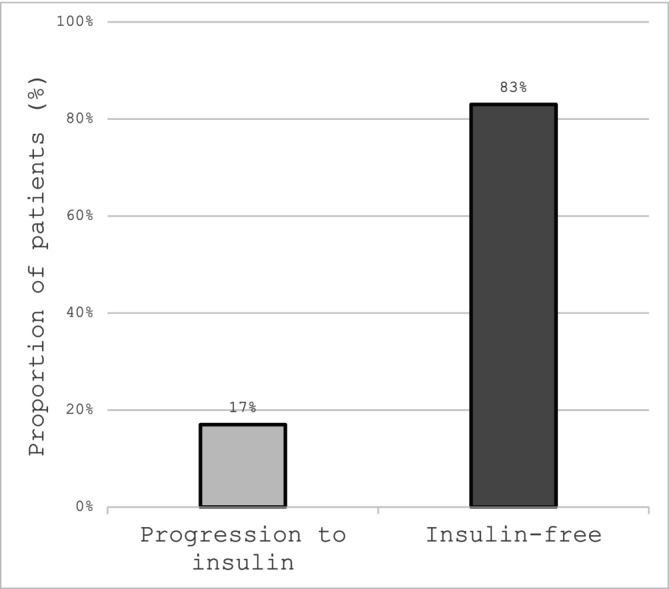
Progression to insulin therapy among patients commenced on Teplizumab at a median follow‐up period of 197 days (*n* = 42).

Median follow‐up time was 197 days after the administration of the first dosage. Seven patients (17%) commenced insulin within a median of 293 days (range 98–374) (Figure 1); data on the length of time for which the other 35 patients remained insulin‐free was not available. No episodes of diabetic ketoacidosis (DKA), abnormal liver function, or anaphylaxis were recorded. Outcome data for T1D diagnosis, rash, and lymphopaenia were unavailable because of small sample sizes and privacy restrictions.

### 5.3 Paediatric versus adult sub‐analysis

Of the 42 patients initiated on Teplizumab, 31 were children or adolescents (aged 8–17 years) and 11 were adults (aged ≥18 years). Among the paediatric group, 5 (16%) progressed to insulin therapy after a median of 308 days (range 98–374), whilst 2 (18%) in the adult group commenced insulin after a median of 170 days (range 170–269). Characteristics of the paediatric and adult sub‐cohorts are presented in Appendix A (Tables A1 and A2).

## 6 CONCLUSIONS

In this real‐world analysis of outcomes in 42 presymptomatic (stage 2) pre‐T1D treated patients with Teplizumab, only 17% of individuals progressed to insulin initiation within approximtely the first year, broadly consistent with clinical trial data. These data support its ability to (immuno)modulate the natural history of T1D and improve long‐term health outcomes.

The median age of our cohort was 12 years, younger than the global median age of symptomatic (Stage 3) diagnosis (~24 years).^8^ As ~75% of individuals with Stage 2 T1D progress within 4–5 years,^9^ many develop symptomatic disease during late adolescence or early adulthood, when maintaining optimal glycaemic control can be challenging due to the lifestyle demands of insulin therapy and dietary management. Early metabolic control has been shown to reduce complication rates decades later.^9^ Delaying progression to symptomatic T1D with agents such as Teplizumab may therefore improve quality of life and reduce long‐term disease burden at a pivotal stage of development.

Pragmatic, scalable strategies are essential to identify individuals eligible for early intervention. The Fr1da study in Germany demonstrated that population‐based islet autoantibody screening in children enables early identification of pre‐symptomatic T1D. Children diagnosed through screening had lower HbA1c, reduced DKA incidence, lower insulin requirements, and higher fasting C‐peptide at diagnosis compared with unscreened peers.^10^ Building on this, the EarLy Surveillance for Autoimmune (ELSA) study in the UK screened nearly 25,000 children aged 3–13 years and found 1.78% to be autoantibody‐positive, including 105 with stage 1 and 31 with stage 2 T1D. High compliance with sample return, confirmatory testing, and education suggests this dried‐blood‐spot approach is feasible at a national level.^11^ A similar framework is being piloted in Italy through the D1Ce Screen study, which aims to screen >5000 children for both autoantibodies and HLA risk genotypes.^12^ Collectively, these initiatives demonstrate that large‐scale childhood screening is achievable and provide critical infrastructure for the equitable deployment of disease‐modifying therapies such as Teplizumab, while informing cost‐effectiveness and implementation models.

Our observed ~one‐year progression rate (17%) is higher than that reported in the landmark TN‐10 trial by Herold et al., where 7% of Teplizumab‐treated participants progressed to Stage 3 T1D compared with 44% in the placebo group.^13^ Although demographic features such as median age, paediatric representation, and sex distribution are comparable between cohorts, baseline glycaemic status differed notably. Our cohort had a median HbA1c of 5.7% (range 4.9%–7.3%) versus 5.2% (range 4.9%–5.4%) in TN‐10, suggesting a more dysglycaemic population at higher risk of progression. The broader HbA1c range in our analysis may also reflect greater heterogeneity in early metabolic control. Differences in attrition rates and dosing details, unavailable on the TriNetX platform, could further contribute to this discrepancy. Additionally, the use of coded electronic health record data introduces potential misclassification, particularly for insulin initiation. Finally, although our results are directionally consistent with evidence from randomised controlled trials, they should be regarded as interim given the absence of longer‐term follow‐up at this stage.

When stratified by age, paediatric and adult outcomes were comparable, consistent with findings from TN‐10, in which no significant age‐related difference in efficacy was observed. Similarly, a clinical review by the U.S Food and Drug Administration (FDA) reported no efficacy difference between children and adolescents, although adult‐specific analyses were limited by small numbers.^14^ In newly diagnosed T1D, the PROTECT phase 3 trial confirmed C‐peptide preservation and reduced insulin requirements in paediatric participants.^7^ Collectively, these data suggest that Teplizumab's benefits extend across age groups, though earlier intervention, typically in younger individuals, may confer greater β‐cell preservation.

Longer‐term follow‐up data provide valuable insight into the sustained effects of Teplizumab beyond the first year. Herold et al. demonstrated persistent separation of Kaplan–Meier curves up to 3 years, with annualised progression rates of 14.9% in the treatment group versus 35.9% in placebo.^13^ Subsequent analyses have shown that benefits can extend beyond 5 years,^15^ and meta‐analyses report larger between‐group differences in insulin use at 24 months compared with 12 months.^16^ Each additional year free from clinical T1D represents a meaningful reduction in lifetime glycaemic burden and downstream complication risk,^3^ reinforcing the importance of early intervention once stage 2 disease is identified.

Clinical trial data consistently demonstrates preserved C‐peptide in Teplizumab‐treated patients, reflecting better β‐cell preservation. HbA1c results are less consistent, with some reporting modest reductions^5^ and others no significant change.^16^ C‐peptide thus appears the more robust marker of pancreatic function and treatment efficacy. We were unable to assess changes in HbA1c or C‐peptide in our cohort. Unfortunately, autoantibody and glycaemic data were available for fewer than 10 patients in our cohort; the much smaller sub‐cohort does, however, demonstrate islet antibody positivity (GAD‐65 and Zinc Transporter‐8) and dysglycaemia, aligning with established criteria for Teplizumab initiation.

Previously reported adverse events include rash, lymphopenia, liver dysfunction, and cytokine release syndrome.^6^, ^7^ While generally more favorable than typical immunomodulation therapy, published follow‐up remains limited to ~2 years. Longer‐term safety surveillance is required. The short follow‐up period (<1 year) reflects the drug's novelty, and electronic health record analyses remain susceptible to coding inconsistencies and missing data. Our study was therefore underpowered to assess rarer outcomes, such as DKA or severe ADEs.

Adults and caregivers of children who have received Teplizumab generally report positive treatment experiences, citing its potential to delay diabetes onset and improve quality of life.^17^ However, equitable access depends on identifying individuals with stage 2 disease, and large‐scale implementation of screening remains a major barrier. Awareness of such screening may be restricted to specialists or even sub‐specialists, and there is a need to encourage more widespread health care professional engagement and education around early T1D screening. Motivations for screening commonly include understanding personal risk, preventing diabetic ketoacidosis, delaying onset of Type 1 diabetes, and contributing to research knowledge. Despite the encouraging data around Teplizumab, many participants still eventually progress to Stage 3 T1D and require continuous lifestyle vigilance and frequent glucose monitoring.

Qualitative research exploring perceptions of disease‐modifying therapies (DMTs) similarly reveals divergent attitudes between adults and caregivers. Adults with T1D tend to view DMTs favorably as opportunities to reduce daily management burden, whereas caregivers of children often express caution, perceiving β‐cell preservation as a temporary reprieve that may prolong uncertainty rather than provide reassurance.^18^ Together, these findings underscore the importance of transparent communication about expected benefits, realistic timelines, and psychosocial impacts when integrating DMTs such as Teplizumab into routine diabetes care.

This study is among the first to evaluate Teplizumab use in real‐world clinical practice across multiple healthcare organisations. Our preliminary findings add to the growing body of clinical trial evidence that Teplizumab significantly delays T1D onset in young people. Future research should address optimal strategies for identifying at‐risk populations in clinical practice, long‐term efficacy, robust monitoring of safety, and ensuring universal and equitable accessibility. Despite these encouraging findings, interpretation is limited by the modest cohort size, incomplete biochemical data, and reliance on coded electronic health record entries, which may underestimate events such as insulin initiation or adverse effects. The short follow‐up period precludes assessment of longer‐term durability, β‐cell function, or safety beyond 1 year of treatment. Ongoing real‐world surveillance and register‐based analysis will therefore be important to confirm the durability of response, clarify optimal dosing strategies, and monitor for late adverse events as clinical adoption ensues.

## FUNDING INFORMATION

This research received no specific grant from any funding agency in the public, commercial, or not‐for‐profit sectors.

## CONFLICT OF INTEREST STATEMENT

S.M. has nothing to declare. M.A. receives a fellowship from the Novo Nordisk UK research foundation and JDRF. R.A.M. has received honoraria from Procter & Gamble, Viatris, Eli Lilly, and Sanofi for educational meetings and investigator‐led funding from Procter & Gamble. D.J.C. has received investigator‐initiated grants from Astra Zeneca and Novo Nordisk, support for education from Perspectum with any financial remuneration from pharmaceutical company consultation made to the University of Liverpool and serves as the Topic Advisor for Type 2 Diabetes medications for The National Institute for Health and Care Excellence (NICE), UK. U.A. has received honoraria from Procter & Gamble, Viatris, Grunenthal, Eli Lilly, Theras, Daiichi Sankyo and Sanofi for educational meetings and funding for attendance to an educational meeting from Sanofi and Daiichi Sankyo. U.A. has also received investigator‐led funding by Procter & Gamble and is a council member of the Royal Society of Medicine's Vascular, Lipid & Metabolic Medicine Section.

## Data Availability

Data used in this study was collected solely from the TriNetX network (https://trinetx.com). This data is not publicly available due to privacy restrictions in place. However, accredited researchers registered with TriNetX might request permission to access data via TriNetX. This may require a data‐sharing agreement and may incur data access fees.

## APPENDIX A RESULTS FROM PAEDIATRIC AND ADULT SUB‐GROUP ANALYSIS

**TABLE A1:** Demographic and clinical characteristics of children and adolescent patients at baseline (*n* = 31).

Characteristic	Teplizumab (*n* = 31)	Range (min, max)
Demographic details		
Age at index, years (Median, IQR)	12 (2)	8, 17
Follow up, days (Median, IQR)	316 (433)	
Male sex, no. (%)	20 (69%)	
Race or ethnic group, no. (%)		
White	21 (72%)	
Hispanic or Latino	<10	
Asian	<10	
American Indian or Alaska Native	<10	
Black or African American	0	
Anthropometric details		
Body mass index, kg/m^2^ (median, IQR) (*n* = 15)	20.4 (3.62)	16.2, 25.6
Body surface area, m^2^ (median, IQR) (*n* ≤ 10)	1.55 (0.25)	0, 174
Biochemistry		
Glycated haemoglobin, % (Median, IQR) (*n* = 20)	5.7 (0.1)	4.9, 6.6
Glycated haemoglobin, mmol/mol (Median) (*n* = 20)	39	30, 49
Oral glucose tolerance test (OGTT), mg/dL (median, IQR) (*n* ≤ 10)	122 (23)	122, 145
C‐peptide, ng/mL (median, IQR) (*n* ≤ 10)	1.89 (0.43)	1.57, 6.6
T1D associated autoantibodies, IU/mL (median, IQR) (*n* ≤ 10)		
GAD‐65	6 (84)	6, 90
IA‐2	10.2 (0)	10.2, 10.2
Insulin‐Ab	0 (0)	0, 0.05
Zt‐8	0.14 (0.14)	0, 174

**TABLE A2:** Demographic and clinical characteristics of adult patients at baseline (*n* = 11).

Characteristic	Teplizumab (*n* = 11)	Range (min, max)
Demographic details		
Age at index, years (Median, IQR)	22 (10)	18, 55
Follow up, days (Median, IQR)	332 (550)	
Male sex, no. (%)	10 (91%)	
Race or ethnic group, no. (%)		
White	10 (91%)	
Hispanic or Latino	<10	
Asian	<10	
American Indian or Alaska Native	<10	
Black or African American	0	
Anthropometric details		
Body mass index, kg/m^2^ (median, IQR) (*n* = 15)	5.8 (0.5)	5.3, 7.3
Body surface area, m^2^ (median, IQR) (*n* ≤ 10)	5	5, 5
Biochemistry		
Glycated haemoglobin, % (Median, IQR) (*n* = 20)	5.8 (0.5)	5.3, 7.3
Glycated haemoglobin, mmol/mol (Median) (*n* = 20)	39	34, 56
Oral glucose tolerance test (OGTT), mg/dL (median, IQR) (*n* ≤ 10)	No data available	—
C‐peptide, ng/mL (median, IQR) (*n* ≤ 10)	1 (0.8)	1, 1.8
T1D associated autoantibodies, IU/mL (median, IQR) (*n* ≤ 10)		
GAD‐65	18.6 (87.4)	18.6, 106
IA‐2	8.1 (0)	8.1, 8.1
Insulin‐Ab	No data available	—
Zt‐8	No data available	—
